# Reduced multimodal integration of memory features following continuous theta burst stimulation of angular gyrus

**DOI:** 10.1016/j.brs.2017.02.011

**Published:** 2017

**Authors:** Yasemin Yazar, Zara M. Bergström, Jon S. Simons

**Affiliations:** a Department of Psychology, University of Cambridge, Cambridge, UK; b Behavioural and Clinical Neuroscience Institute, University of Cambridge, UK; c School of Psychology, University of Kent, Canterbury, UK

**Keywords:** Parietal lobe, Memory, Recollection, Source monitoring, Brain stimulation

## Abstract

**Background:**

Lesions of the angular gyrus (AnG) region of human parietal cortex do not cause amnesia, but appear to be associated with reduction in the ability to consciously experience the reliving of previous events.

**Objectives/Hypothesis:**

We used continuous theta burst stimulation to test the hypothesis that the cognitive mechanism implicated in this memory deficit might be the integration of retrieved sensory event features into a coherent multimodal memory representation.

**Methods:**

Healthy volunteers received stimulation to AnG or a vertex control site after studying stimuli that each comprised a visual object embedded in a scene, with the name of the object presented auditorily. Participants were then asked to make memory judgments about the studied stimuli that involved recollection of single event features (visual or auditory), or required integration of event features within the same modality, or across modalities.

**Results:**

Participants' ability to retrieve context features from across multiple modalities was significantly reduced after AnG stimulation compared to stimulation of the vertex. This effect was observed only for the integration of cross-modal context features but not for integration of features within the same modality, and could not be accounted for by task difficulty as performance was matched across integration conditions following vertex stimulation.

**Conclusion:**

These results support the hypothesis that AnG is necessary for the multimodal integration of distributed cortical episodic features into a unified conscious representation that enables the experience of remembering.

## Highlights

• We used brain stimulation to test role of angular gyrus in episodic memory.
• Participants made memory judgments that required integration of event features.
• Retrieval of integrated multimodal features reduced after angular gyrus stimulation.
• Within modality integration unaffected, and task difficulty explanation ruled out.
• Angular gyrus integrates multimodal features into unified memory representation.

## 1 Introduction

Growing evidence indicates that the angular gyrus (AnG) region of lateral parietal cortex is critical for subjective aspects of contextual recollection that draw on the conscious experience of reliving previous events. Neuropsychological studies of patients with AnG lesions have demonstrated selective impairment on memory measures that emphasize experiential qualities of remembering, such as spontaneous autobiographical recall, ‘remember’ responses on remember/know tasks, and recollection confidence ratings [1], [2], [3]. Similarly, neuroimaging studies have reported enhanced AnG activity in healthy volunteers associated with assessments of recollective experience [4], [5], [6]. Recent TMS studies provide further evidence: targeting left AnG, Sestieri et al. [7] reported altered response bias in source memory attributions, indicating a role in the weighing of relevant retrieved information. Similarly, Yazar et al. [8] found that disrupting left AnG reduced participants' confidence in their contextual recollections.

Although a causal relationship between AnG and recollection has been identified, the information processing operations subserved by this region that enable the conscious experience of remembering remain unresolved. Here, we examine the proposal that during episodic memory retrieval, AnG supports processes that integrate retrieved event features of different modalities that are distributed across cortical regions into a coherent multimodal mnemonic representation [6], [9], [10]. Its position at the intersection between sensory association areas that are important for unimodal feature binding [11] makes AnG an ideal candidate for a role in the integration of cross-modal features to form a unified episodic memory representation. Moreover, its anatomical connectivity supports rich interactions with fronto-temporal and medial cortical regions associated with memory, such as hippocampus and precuneus [12]. Consistent with this proposal, Bonnici et al. recently observed neuroimaging evidence of greater AnG activity during retrieval of integrated multimodal episodic memories compared with unimodal episodic memories [9].

If AnG does indeed mediate multimodal integration during recollection then, in addition to being engaged during retrieval of multimodal memories, the region should be necessary for accurate performance on memory tasks that are dependent on such binding. Previous neuropsychological and TMS studies used memory tasks that tested retrieval of one feature at a time, such as whether a male or female speaker had read a word aloud [3], [8], [13], [14], which is insufficient to address this question. To test the hypothesis that AnG is necessary for integrating multisensory episodic memory features, we developed a task in which participants were asked to remember previously studied audiovisual stimuli and make recollective judgments that differed in the kind of episodic feature integration required.

The simplest form of retrieval involved recollection of only a single episodic detail, similar to previous studies, and no impairment following AnG TMS stimulation was expected. The key conditions of interest were memory judgments that required the integration of event features either within the same modality, or across modalities. A role for AnG in integration regardless of modality would predict reduced performance following AnG stimulation in both conditions. If the role of AnG during recollection is, however, specifically to bind multimodal memory features into a conscious representation that enables the subjective ‘reliving’ of an event, as the neuroimaging results reported by Bonnici et al. [9] predict, then TMS disruption should reduce performance selectively when cross-modal integration is required.

## 2 Material and methods

### 2.1 Participants

Twenty-four healthy, right-handed, native English speakers (15 female), aged 21–34 years (M = 25.13, SD = 3.88), were recruited from volunteer panels. Participants were each tested on two separate occasions with site of stimulation (AnG or vertex) manipulated as a within-subjects variable. Participants were randomly assigned to receive AnG or vertex stimulation in their first session. All subjects had normal hearing and normal or corrected-to-normal vision and were screened for possible contra-indications to TMS. Subjects gave informed consent to participate in the study in a manner approved by the University of Cambridge Human Biology Research Ethics Committee, and were reimbursed for their participation. Data from one participant had to be excluded because of failure to attend the second session, leaving 23 subjects who completed both sessions.

### 2.2 Stimuli

A total of 248 audiovisual stimuli were used, each comprising a natural scene picture, an object picture, and the spoken word that referred to the object. Scenes were selected from the database at http://image-net.org. All scene pictures depicted daylight settings, and were selected such that an object could be embedded into the left or right side of the scene and could have a spatial relation to another object in the scene, such as being on top of or underneath something else. The objects were selected from the Hemera Photo-Objects 5000 CD and from ‘Google Images’. The 248 words that referred to these objects were between three and twelve letters long, with a Kucera-Francis frequency of 20–100, familiarity ratings of 300–700, and concreteness and imageability ratings of 400–800 (chosen from the Medical Research Council Psycholinguistics database at http://tinyurl.com/mrc-database). All word stimuli were recorded in both English and Scottish accents by one female and one male speaker (resulting in four versions of each word) with the audio editor *Audacity*^*®*^ *(*http://audacity.sourceforge.net/).

**Fig. 1:**
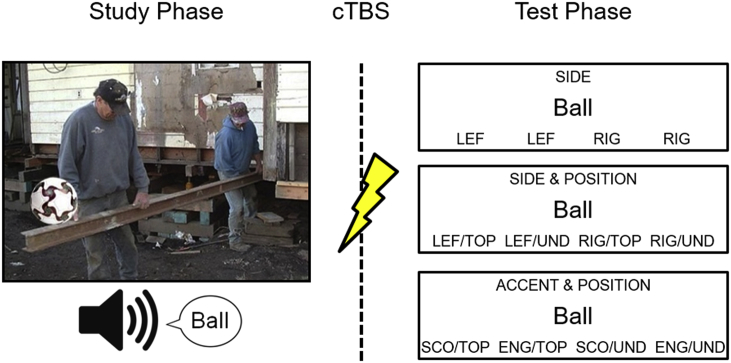
Schematic representation of the experiment. In each study phase trial, an object (in this case, a ball) was presented on the left or right of a scene, on top of or underneath another salient object. Concurrently, the name of the object was presented auditorily in a male or female voice, spoken in a Scottish or English accent. Following cTBS targeting vertex or angular gyrus, the test phase was administered. Examples of the three source memory conditions are displayed (top: single source, middle: within-modal source, bottom: cross-modal source). See text for further details.

To create the source features for the different study conditions, each scene picture was edited using *Microsoft Paint*. The embedded object was inserted either on the left or on the right side of the scene picture and was located either on top of or under another salient object in the scene. Four versions of each visual stimulus were created, with the object either on the left side and on top of something else in the scene, on the left side and under something else in the screen, on the right side and on top of something else in the scene or on the right side and under something else in the scene. The word was spoken either by a female voice or by a male voice, speaking in either an English or a Scottish accent. The source test conditions were created in the following manner: visual and auditory stimuli could be tested for *single source* features (position: top/under; side: left/right; gender: male/female; accent: English/Scottish); or combined to test for *within-modality source* features (side *and* position: left/top, left/under, right/top, right/under; accent *and* gender: Scottish/male, Scottish/female, English/male, English/female); or combined to test for *cross-modal source* features (gender *and* position: male/top, female/top, male/under, female/under; accent *and* position: Scottish/top, English/top, Scottish/under, English/under; gender *and* side: male/left, female/left, male/right, female/right; accent *and* side: Scottish/left, English/left, Scottish/right, English/right); see Fig. 1 for an example. These combinations resulted in 24 different counterbalancing formats that were rotated across participants. Order of the conditions was pseudo-randomised with no more than three consecutive trials having the same source feature conditions.

### 2.3 Procedure

All participants completed the same memory tasks with different stimuli on two different occasions, one experimental (AnG stimulation), and one control session (vertex stimulation), with session order counterbalanced across participants. The two sessions were three days apart, scheduled at the same time of day. On both occasions participants underwent the same procedure: practice run, study phase, continuous theta-burst stimulation (cTBS) procedure, and test phase. At the beginning of the first session, each individual's resting motor threshold was assessed (see cTBS procedure). Participants were then familiarised with examples of the visual and auditory stimuli and instructed about the different source conditions. Once subjects were familiar with the task, a practice session was completed.

In 72 study phase trials, a fixation cross was presented for 250 ms, followed by a 3500 ms presentation of the visual stimulus. Concurrently, the auditory stimulus was presented via loudspeakers. Subjects were prompted to make a pleasantness judgment about the object by pressing one key for ‘pleasant’ and another key for ‘unpleasant’. Following cTBS, in 108 test phase trials, after a 500 ms fixation cross, a written word was presented in the middle of the screen and participants were instructed to decide whether the word had been studied or was a new word. If participants responded ‘new’, the next stimulus was then presented, otherwise they were asked to make a source judgment. The type of source condition (single source, within-modal source, cross-modal source) was displayed at the top of the screen, the target word was presented in the middle of the screen, and at the bottom of the screen four response options were displayed.

The test phase comprised four blocks of each condition (four single source blocks, four within-modality blocks, four cross-modality blocks). All blocks were presented in random order and comprised nine trials each (six old items, three new items per source block, presented in random order). In single source trials, each response option was displayed twice (to match response demands with the other conditions), and subjects were encouraged to spread their responses (e.g. in a *gender* block, the response options would be ‘MAL’, ‘MAL’, ‘FEM’, ‘FEM’; for male and female), whereas the dual source judgments had four different response options (e.g. in an *accent & gender* block the response options would be ‘SCOT/MAL’, ‘SCOT/FEM’, ‘ENGL/MAL’, ‘ENGL/FEM’).

### 2.4 cTBS procedure

At the beginning of the first session, each subject's individual resting motor threshold was assessed for the right first dorsal interosseous hand muscle. In both sessions, following the study phase, the subject's head was co-registered to their brain image via previously identified anatomical landmarks using the neuronavigation system software Brainsight (Rogue Research, Canada). To guide frameless stereotaxy we used target centre of mass MNI coordinates described in a previous meta-review of the parietal lobe and memory [15] for AnG (−43, −66, 38) and a probabilistic anatomical atlas [16] for vertex (0, −15, 74). A standard conditioning cTBS protocol was then delivered with three pulses at 50 Hz, repeated every 200 ms for 40 s at 70% resting motor threshold to either left AnG or to vertex [17]. Stimulation was delivered via a Magstim Rapid^2^ (Whitland, UK) with a standard 70 mm diameter figure-of-eight coil.

## 3 Results

Mean recognition accuracy (correctly recognised old items and rejected new items) and source recollection accuracy (conditionalized on correct recognition) for each condition are displayed in Table 1, along with reaction times. Planned comparisons tested for the presence of cTBS-induced performance impairments following AnG versus vertex stimulation. Effect sizes were calculated using Cohen's d or partial eta-squared (η_p_^2^), as appropriate.

**Table 1:** Participants' mean (and standard deviation) accuracy on the recognition and source memory tasks following stimulation to vertex and AnG.

Memory Measure	Vertex		AnG	
	Score	RT	Score	RT
Hits	0.85 (0.12)	2867 (1075)	0.84 (0.14)	2998 (1357)
Correct rejections	0.92 (0.09)	3533 (1980)	0.90 (0.09)	3759 (2360)

Single source recollection	0.76 (0.11)	2503 (1130)	0.72 (0.13)	2750 (1508)
Within-modal recollection	0.52 (0.15)	3894 (2066)	0.54 (0.14)	4229 (2375)
Cross-modal recollection	0.54 (0.11)	4684 (2692)	0.48 (0.14)	5085 (2951)

Note: AnG = angular gyrus, RT = reaction time, old/new recognition calculated as correctly recognized old items, source recollection conditionalized on correct recognition.

As expected based on previous neuropsychology and TMS studies, there was no reduction in either the hit rate, t(22) = 0.295, *p* = 0.771, d = 0.061, or correct rejection rate, t(22) = 0.610, *p* = 0.548, d = 0.127, or their associated RTs, t(22) < 0.95, p > 0.353, d < 0.104, after AnG stimulation compared to vertex stimulation. Also as predicted, single source performance was not significantly reduced after AnG stimulation, t(22) = 1.475, *p* = 0.154, d = 0.308.

**Fig. 2:**
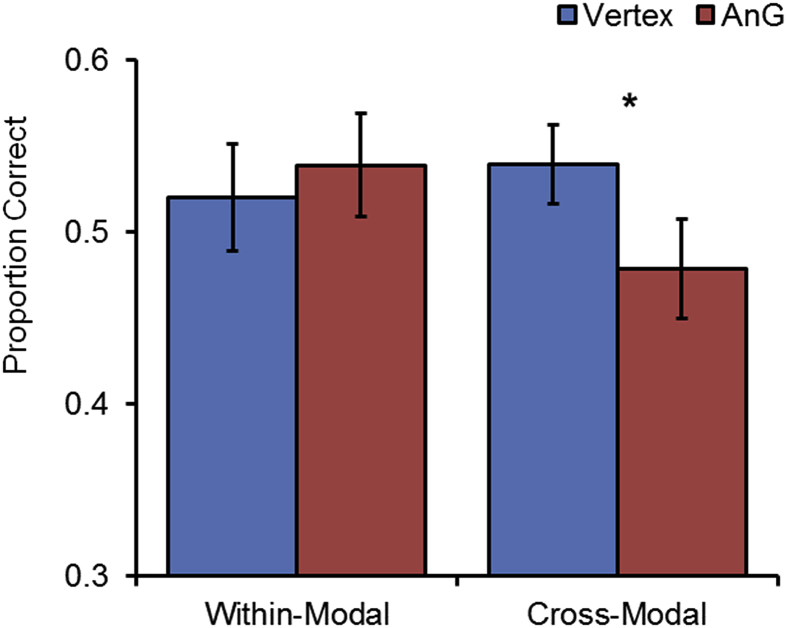
Source memory accuracy of participants after cTBS targeting vertex or angular gyrus (AnG) in conditions that required the integration of event features either within the same modality, or across modalities. A selective impairment was observed in cross-modal source accuracy following AnG stimulation.

Turning to the integration conditions (see Fig. 2), there was no difference in source accuracy for within-modality judgments after AnG stimulation compared to vertex stimulation, t(22) = 0.483, *p* = 0.634, d = 0.101. However, consistent with predictions of the multimodal integration hypothesis, source accuracy for cross-modal trials was significantly reduced following AnG stimulation compared to vertex stimulation, t(22) = 2.329, *p* = 0.029, d = 0.486. The interaction between integration condition (within-modal vs cross-modal) and stimulation site (AnG vs vertex) exhibited a trend towards significance, F(1,22) = 3.178, *p* = 0.088, η_p_^2^ = 0.126. As the multimodal integration hypothesis predicts a specific reduction in cross-modal accuracy with within-modal accuracy unaffected (rather than a crossover interaction), a “selective effect” ANOVA model was applied to the data [18], which confirmed the existence of a significant ordinal interaction, with AnG stimulation affecting cross-modal more than within-modal source accuracy, F(1,22) = 5.813, *p* = 0.025, η_p_^2^ = 0.209.

The cross-modal source impairment could not be attributed to task difficulty, as within-modal and cross-modal source performance were matched following vertex stimulation, t(22) = 0.667, *p* = 0.512, d = 0.139. Site of stimulation had no significant effect on source judgment RTs, t(22) < 1.463, *p* > 0.158, d < 0.142.

## 4 Discussion

This experiment investigated the multimodal integration of memory features as a putative information processing operation subserved by AnG that might underlie the conscious experience of reliving previous events. We tested the hypothesis that AnG is necessary for multimodal integration during recollection by examining the effect of continuous theta-burst stimulation (cTBS) on performance of memory tasks that involved the retrieval of single event features, or required the integration of features within the same modality, or across modalities. The ability to recollect context features from across multiple modalities was significantly reduced after AnG stimulation compared to stimulation of a vertex control site. Stimulation made no difference to retrieval that involved integration of features within the same modality, a selective cross-modal binding effect that cannot be attributed to differences in task difficulty because performance was matched across integration conditions following vertex stimulation. These results support the proposal that AnG is necessary for integrating distributed sensory event features into a unified representation that supports the experience of remembering [6], [9], [10].

The present findings corroborate evidence that AnG is an integral part of a core recollection network that also includes frontal, medial temporal, and medial parietal regions [19], [20], and provide further constraints on theorising about the kind of processes for which the AnG node of this network might be responsible. The proposed role in integrating multimodal memory features [9] fits with considerations of AnG as an integrative hub that contributes to a wide variety of cognitive demands which rely on the coordination of multiple distributed representations, including associative semantics, attention, reasoning, and social cognition [12], [21], [22]. The location of AnG at the intersection between temporal, parietal, and occipital cortices, i.e. between visual, spatial, auditory, and somatosensory association areas, and its rich anatomical connectivity via a number of major white matter tracts and bundles, place it in an ideal position to be a “high-level supramodal integration area in the human brain” [23]. However, several other brain regions have also been identified as potential “convergence zones” [21], including another key node of the core recollection network, the hippocampus. Various integrative functions in episodic memory have been ascribed to the hippocampus, including the binding of episodic memory features via pattern completion mechanisms [24], [25]. The attribution of apparently similar memory functions to two distinct brain regions leads to the question of how hippocampal and parietal roles might be differentiated.

We have proposed that the binding process mediated by AnG may be distinguished from that of the hippocampus in terms of the spatial framework within which the integrated memory representation is constructed. Evidence from studies of spatial navigation suggest that parietal lobe regions may support *egocentric* spatial processing rather than the *allocentric* “cognitive map” spatial functions that characterize the hippocampus [26], [27], [28], [29]. For example, Ciaramelli et al. [28] observed that patients with parietal lobe lesions were impaired on egocentric spatial memory tasks such as landmark sequencing and route navigation, which healthy volunteers reported accomplishing by imagining their own body position with respect to relevant landmarks. Parietal patients were unimpaired on spatial tasks that were considered to rely on an allocentric strategy, such as imagining a map on which the various landmarks were located. This pattern contrasts with evidence from patients with selective hippocampal lesions, who are impaired on tasks assessing allocentric but not egocentric spatial memory [30]. Consistent with the notion of impairment in egocentric aspects of memory following parietal lobe damage, Berryhill et al. [31] observed that when patients with parietal lesions recalled past autobiographical events or imagined novel constructed experiences, they were less likely to represent themselves in the scenes that they created, reporting fewer details about their thinking, their emotional states and their own actions during their narratives.

Neuroimaging evidence, with its greater anatomical specificity compared to neuropsychological lesions, indicates that egocentric spatial processing may be subserved particularly by medial parietal regions such as the precuneus [32]. For example, Rosenbaum et al. [33] found that when healthy volunteers were scanned using fMRI while undertaking the landmark sequencing and route navigation spatial memory tasks mentioned above, significant activity was observed in left medial parietal regions around the precuneus. In a similar vein, Zaehle et al. [27] asked participants to make spatial judgments in response to verbal descriptions of object relationships that were framed either with respect to participants themselves, or without any body-centered references. Egocentric spatial coding engaged the precuneus, whereas allocentric coding involved the hippocampus. Consistent with this distinction, Wolbers et al. [34] observed activation in the precuneus when participants performed a task in a virtual environment that involved keeping track of the positions of surrounding objects relative to their own bodies. The precuneus may thus be critical for providing an egocentric spatial framework, which may then be utilised by AnG in constructing an integrated episodic recollection [35]. Strong anatomical connectivity between the precuneus and AnG, via the occipito-frontal fascicle, has been identified by diffusion-tensor based segmentation and tractography studies [36], consistent with the idea that a function of the AnG might be to integrate multimodal memory features within an egocentric framework into the kind of first-person perspective representation that enables the subjective re-experiencing of an event [37].

Several other theoretical accounts have been proposed concerning the possible contribution of AnG to episodic memory, such as a temporary storage buffer in which retrieved information can be accumulated to facilitate decision-making processes [15], or a role in the bottom-up capturing of attention by retrieved information [38]. Both alternative accounts are compatible with aspects of the present data, but neither appears able to explain the selective reduction in cross-modal but not within-modal source accuracy. The two conditions were designed to be matched in the amount of information to be retrieved, both requiring the integration of two different event features (e.g., side and position for within-modal judgments, or gender and position for cross-modal judgments). Moreover, it is not clear why cross-modal memories might be more likely than within-modal memories to capture attention, particularly given that the two conditions elicited similar performance levels following vertex stimulation. A number of previous studies have provided other evidence that cannot easily be reconciled with the buffer or attentional perspectives [3], [8], [13], [39], [40], [41]. For example, recent experiments using neuroimaging methods to decode the content of the memory representations supported by AnG indicate that the region may be less sensitive to the amount of information represented or its attentional salience than to the multimodal nature of the retrieved information [6], [9].

To conclude, this experiment found that brain stimulation targeting AnG selectively reduced participants' ability to perform memory judgments that required the integration of auditory and visual information, leaving intact performance on tasks that involved the retrieval of single event features, or required the integration of features within the same modality. This finding is consistent with the results of previous neuroimaging and neuropsychology studies, and can be interpreted in the light of proposals that the role played by AnG in episodic memory may be in integrating the sights, sounds, and smells comprising a previously experienced event into a coherent, multimodal representation during retrieval that enables the rich and vivid reliving of the past.
